# Supplementary material for: The discovery of novel noncoding RNAs in 50 bacterial genomes
Source: Nucleic Acids Res. 2024 Apr 22;52(9):5152–65. doi: 10.1093/nar/gkae248 (PMC11109978; doi:10.1093/nar/gkae248)
Supplement: gkae248_Supplemental_Files [file gkae248_supplemental_files.zip › Narunsky 50 Genomes RNA Motifs Files.zip!/Publication/59-G_aurantiaca/Figures/LRC-59-9-NC_012489_1_IGR_50_1116066_1116603.sto.pdf]

LRC-59-9-NC_012489_1_IGR_50_1116066_1116603.sto.r2r

5′-○○YGUG●R●●●RGGCAA●UGY●●GYU○●Y●GUGUGAR●GCRRGCAAR○○○○○○○○CGA○●●CG●R●A●U●GCGC●○○●GCRGRY●Y●●U●●YYY●UG●CG●G●YRGUGY●G●R●UG●YR●●●G●C●●CG●G●●●●●CR○○●A●●●G●AAR●●●●URYRG●●○●AY○○○○○●G●U●●U●RC○○○○○○○○○○○AC●●YR●●●●CRC●●●G●RRC●○○●●●●●A●○RYYG●●●R●UYG●●C●●G○○●●●RYCRCACAAR●●UG●●●●YGGUGGA●AACU●●●Y●R●Y●YYGCA●R●A●GUGAUGU●●CR●●UGCAA●U●R●●●CY●●●YA●●GCGUUR●●●YRYGACUUGCGCGG●CUCACGR●YRGAG●●ACY●UAC●●CCCCGCUCRGCCCYU●○●C●●●●●YCCGGG○○○○○○CC●G●RA●●●C●Y●●●C●●●●YGY●●YG●C●CACGYY●○YRC●●CAGGAGY●CCG○○○
